# Thermal Conductivity Characterization of Thermal Grease Containing Copper Nanopowder

**DOI:** 10.3390/ma13081893

**Published:** 2020-04-17

**Authors:** Haneul Kang, Hyunji Kim, Jihye An, Siyeon Choi, Jinho Yang, Hyomin Jeong, Sunchul Huh

**Affiliations:** 1 Department of Energy and Mechanical Engineering, Graduate School of Gyeongsang National University, Tongyeonghaean-ro 2, Tongyeong-si 53064, Korea; hnkang@gnu.ac.kr (H.K.); hjkimgnu@gnu.ac.kr (H.K.); jhan@gnu.ac.kr (J.A.); 2 Department of Mechanical Engineering System, KOPO Deajeon-si Polytechnic Campus, Deajeon-si 34503, Korea; csy0813@kopo.ac.kr; 3 Department of Computer Aided Machinery, KOPO Iksan-si Polytechnic Campus, Iksan-si 54567, Korea; bestgno@kopo.ac.kr; 4 Department of Energy and Mechanical Engineering, Gyeongsang National University, Tongyeonghaean-ro 2, Tongyeong-si 53064, Korea; hmjeong@gnu.ac.kr

**Keywords:** thermal interface materials, nano powder, thermal grease, thermal conductivity, graphene, alumina

## Abstract

As electronic devices and mainboards become smaller, the need for thermal conductive materials having excellent internal heat dissipation is increasing. In this study, nano thermal grease was prepared by mixing in copper nanopowder, which is used as a heat transfer medium in thermal grease, which is a kind of thermal conductive material, with silicon oil. In addition, copper powder was mixed with graphene and alumina, respectively, and the thermal conductivity performance was compared. As a result, the thermal conductivity improved by 4.5 W/m·k over the silicon base, and the upward trend of thermal conductivity increased steadily up to 15 vol. %, and the increasing trend decreased after 20 vol. %. In addition, the increased rate of thermal conductivity from 0 to 5 vol. % and 10 to 15 vol. % was the largest.

## 1. Introduction

As electronics and motherboards become smaller, the power consumption required increases significantly, resulting in a sharp increase in emission power density [1,2,3], This increase in power density is associated with the internal thermal characteristics of the device, which is directly related to the performance and efficiency of the electronic device [4,5,6]. In order to improve the performance of the electronic device, a heat conducting material having excellent internal heat dissipation performance was used. In recent years, the need for heat conductive materials is increasing, and research on high efficiency heat conductive materials having high performance of heat transfer and heat resistance is required. To improve the performance and efficiency of electronic devices, heat sinks must be used to control the heat generated during operation more quickly and effectively [7]. In addition, even if the heat dissipation performance is excellent, the interfacial thermal conductivity is reduced by forming an air layer due to a mismatch between the specific surface and the surface roughness between the device and the heat sink. In general, in order to control heat dissipation characteristics of electronic products with heat generation, a heat conductive material (TIMs: Thermal Interface Materials) is used to reduce heat resistance due to surface roughness and to improve a contact area between the device and the heat sink [8].

An ideal model of this thermal conductive material is applied between the heat sink and the device to remove the air layer present at the interface in order to improve the contact area, thus reducing the thermal contact resistance and enhancing smooth heat transfer. One of the TIMs materials, thermal grease, is used to enhance the thermal conductivity on bonded solid surfaces including between heat sinks. Specifically, it is applied to the heat spreader and the heat sink interface of the central processing unit (CPU) or graphics processing unit (GPU) in order to reduce the thermal contact resistance by removing the air layer and improving the contact area [9,10,11,12].

Generally, thermal grease consists of a polymer material and a ceramic filler material [13,14,15,16]. Silicone is generally used as a base material of thermal grease due to its excellent thermal stability and relatively easy processing [17,18,19]. Ceramic fillers such as copper, which are thermally conductive and electrically resistant, are utilized [20,21,22].

In this study, nano thermal grease was prepared by mixing copper nanopowder used as heat transfer medium with silicon oil. In addition, copper powder was mixed with graphene and alumina, respectively, and thermal conductivity performance was compared and analyzed. The purpose of this study is to investigate the thermal conductivity performance of the copper powder.

## 2. Experiment Equipment and Method

### 2.1. Experiment Preparation

**Figure 1:**
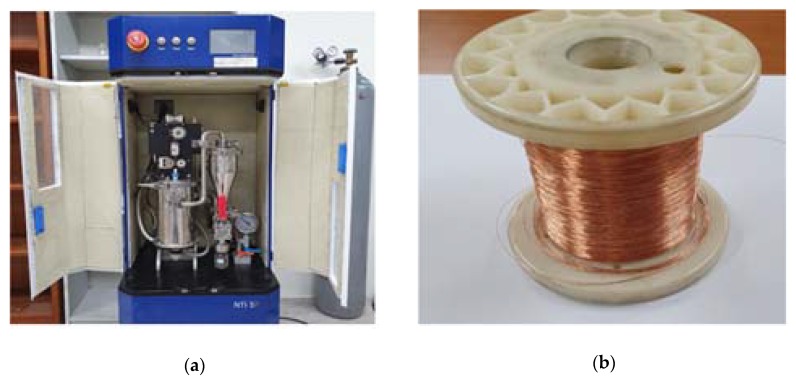
Pursed wire evaporation (PWE) equipment used to manufacture nano powder: (**a**) photograph of PWE equipment and (**b**) photograph of copper metal wire used in PWE equipment.

“A copper powder” sample stored for more than 6 months, was purchased from Nano Technology Company in Busan-si, Korea (purity of 99.9%, average radius size of 100 nm) while “B copper powder” sample stored up to 6 days from the day of direct manufacture, was used for electric wire explosion method. It was prepared using pulsed wire evaporation (PWE). Figure 1a shows a photograph of the Nano-powder manufacturing equipment, (b) presents a photo of the 0.2 mm copper metal wire used in the PWE equipment. PWE equipment utilized NTi 5P model from Korea Nano Technology. PWE is a technique of producing Nano-sized powder via evaporation and condensation process. This is achieved through sublimation of a certain length of metal wire by supplying high density electrical energy to the metal wire in a short time (0.0001 s) [23,24,25].

**Figure 2:**
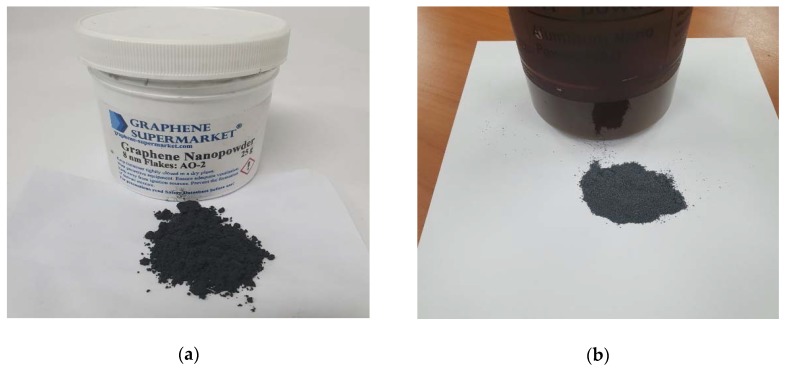
Photograph of nano powder: (**a**) graphene and (**b**) Al₂O₃.

Graphene powder is a commercial product purchased from Junsei Chemical Co., Ltd. In Tokyo, Japan with a purity of 99.9% and a 100 m²/g size of 8 nm. And alumina powder is alumina powder with purity of 99.9% and average particle size of 100 nm. Same as Figure 2a,b. 

Table 1 presents the production conditions of copper Nano powder. PWE can regulate the size of the powder by adjusting the intensity of the voltage applied to the metal wire. At the time of preparation, 2000 stints of 4500 V were conducted to prepare an average of 150 nm powder.

KF-96 was directly used as base silicone oil for Nano thermal grease and was purchased from ShinEtsu in Seoul-si, Korea. without further purification. The commercially available copper powder and the prepared copper powder were formulated using the same production conditions. For uniformity, the thermal grease was mixed only with the commercially available copper powder and the manufactured copper powder was prepared using a similar process.

### 2.2. Nano Thermal Grease Manufactured

**Figure 3:**
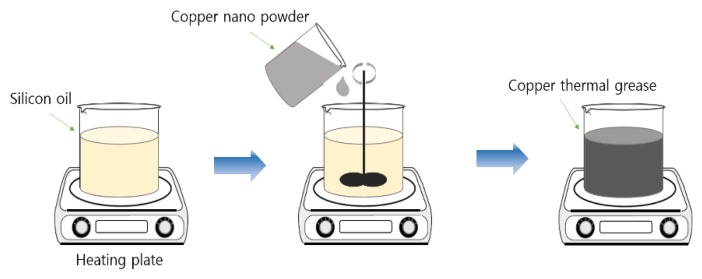
Manufacturing process of nano thermal grease.

Figure 3 shows the process of manufacturing nano thermal grease. Each sample was mixed with copper powder in 100 cc base silicone oil according to the volume percentage, the mixing ratio of the copper powder is shown in Table 2. 

In preparing the thermal grease of graphene, alumina, and copper, each of graphene and alumina was mixed with silicone oil, and then 5 vol. % of copper powder was further mixed. As the mixing ratio increases, the copper nanopowder was not uniformly mixed due to the high viscosity, and each sample was stirred for 30 min at a speed of 300 rpm at 100 °C using a hot plate.

### 2.3. Measuring Equipment

The measurement was performed using SEM equipment. (JSM-6010LA manufactured by JEOL, in Tokyo, Japan). Before the photographing, it was dried in a heating furnace at 100 °C for 8 h as a pretreatment process, and platinum coating was performed.

**Figure 4:**
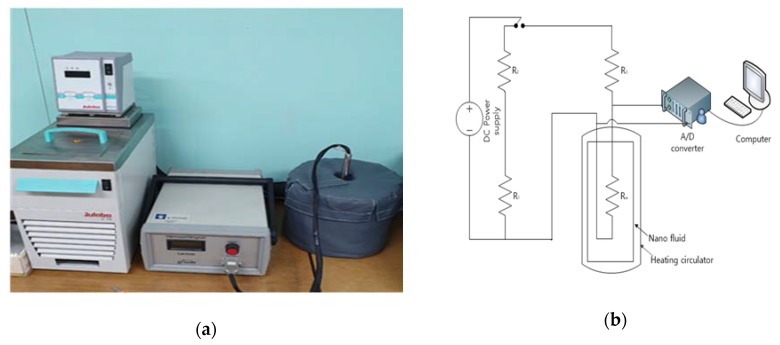
(**a**) Photograph of thermal conductivity meter and (**b**) schematic diagram of hot-wire apparatus.

Figure 4a is a picture of thermal conductivity measurement equipment, KN148 Lambda equipment of Flucon co.,Ltd in Seongnam-si, Korea was used. Figure 4b is a schematic diagram of an experimental apparatus of a hot-wire system. The thermal conductivity was measured using a hot-wire instrument method, which has been widely used to measure the thermal conductivity of a fluid with high accuracy. Based on the calculation of the temperature transport field around the internal metal wires, the thermal conductivity of the fluid using an electric heating element and a resistance thermometer. It is a measuring principle. In addition, the schematic diagram of the equipment shows the sensors, power control devices and computer software.

The heat generated is conducted in the material through the sensor during the rapid drop in temperature of the heating source and the thermal conductivity is calculated from the data of the voltage drop. [26] This method was first introduced by Nagaska and Nagashima in 1981. It is non-destructive and convenient for measuring the thermal conductivity of materials in solid, liquid, powder, and mixed states [27].

## 3. Results

### 3.1. Copper Nano Powder Manufacturing Result

**Figure 5:**
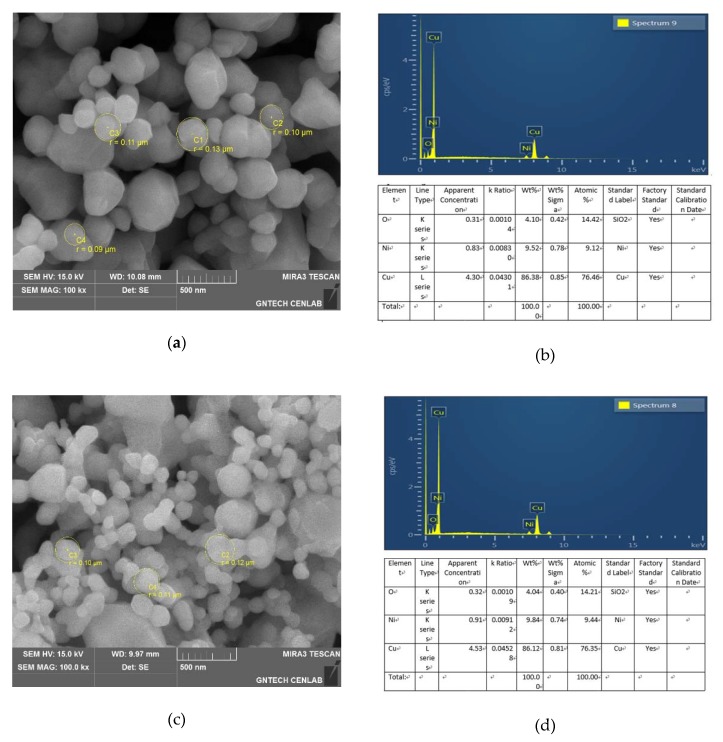
SEM images of (**a**) SEI (Secondary Electron Image) of Cu particles (A), (**b**) EDS (Energy Dispersive Spectrometer) of Cu particles (A), (**c**) SEI of Cu particles (B); (**d**) EDS of Cu particles (B).

Figure 5 is the result of FE-SEM to observe the shape and size of the Cu powder prepared using PWE. Figure 5a is SEI (Secondary Electron Image) of commercially available copper powder, and Figure 5b is the result of EDS (Energy Dispersive Spectrometer). It can be seen from (a) that the average radius is spherical with a diameter of 100nm, and the particle size area ratio is composed of large and small particles of 85:15. As a result of the component analysis, O, Ni and Cu were detected. The cause of detection of O is due to the oxidation process, and Ni is considered to be an impurity such as dust. Figure 5c is SEI of the manufactured copper powder, (d) is EDS result. It can be seen from (c) that the average radius is spherical with a diameter of 100nm, it can be seen that the particle size area ratio is composed of large particles and small particles of 85:15. As a result of the component analysis, O, Ni and Cu were detected. The cause of O and Ni detection is the same as before. As a result, it can be confirmed that impurities other than copper were not produced in the copper nanopowders prepared through (b) and (d).

### 3.2. Thermal Grease Manufacturing Result

**Figure 6:**
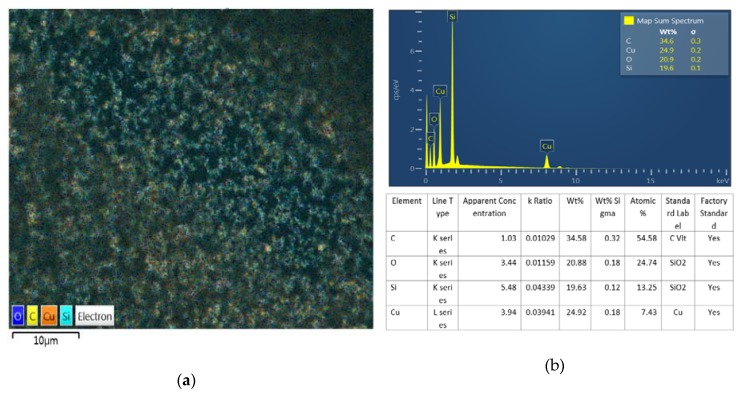
SEM Images of: (**a**) BEI of copper thermal grease and (**b**) EDS of copper thermal grease.

Figure 6a is a BEI (Back scattered Electron Image) photographed using the FE-SEM thermal grease mixed with the prepared copper particles, (b) is the result of the component detection analysis of (a). When electrons collide with electrons like BEI, electrons inside the metal absorb most of the energy and bounce off or bounce. Unlike SEI, electrons emitted out of the sample surface are known to know whether the speed will decrease even after turning the nucleus.

All elements have different atomic nuclei, and the larger the BEI number, the larger the number. This serves to distinguish different elements on the sample.

Therefore, unlike SEI, it is useful to detect information about the composition of the sample. Through this, it means that when manufacturing the thermally conductive grease, the copper nanopowders are not evenly distributed without clumping. In addition, the detection cause of C is a carbon tape used for fixing a sample, O is an oxidation reaction component, Si is a silicon oil, Cu is estimated that copper powder was detected. It means that impurities other than the thermal grease were not generated in the process of preparing the thermal grease.

### 3.3. Thermal Conductivity Characteristics of Manufactured Thermal Grease

**Figure 7:**
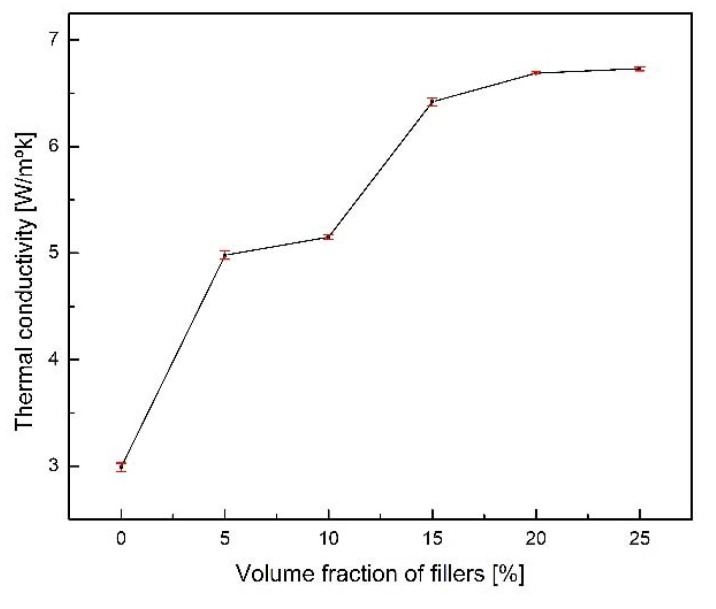
Thermal conductivity of thermal grease of volume fraction of copper powder.

The thermal conductivity of thermal grease is influenced by the particle size distribution of the additive. Cumberland et al. [28] calculated the maximum packing volume fraction of the heterogeneous mixtures of the spheres as a function of the diameter ratio parameter. According to their results, the small volume fraction increases with the packing volume fraction increases until it attains a maximum value and then decreases with further increase after the maximum value. Figure 7 illustrates the results of thermal conductivity versus volume percentage of small copper particles in volume percentage. It can be observed that the thermal conductivity of the thermal grease mixed with copper particles of large and small hybrid size increases with an increase in volume percentage of the small particles and then decreases after a given volume percentage.

The graph shows that the thermal conductivity decreases as the volume fraction of small particles tends to 6.5 ± 0.03 W/m·k at 15% and then increases as the volume percentage increases. This can be attributed by the increase in the thermal conductivity with increasing volume.

**Figure 8:**
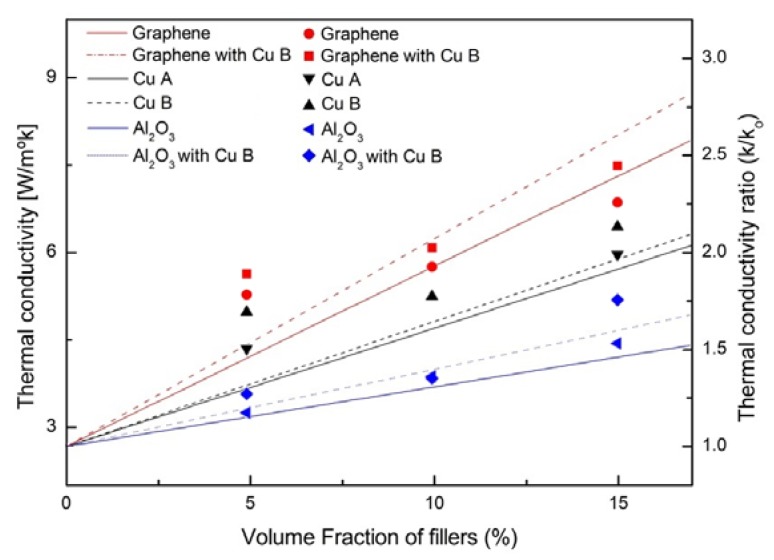
Result of thermal conductivity measurement and thermal conductivity ratio each case.

Figure 8 shows the thermal conductivity measurement results and thermal conductivity ratio in regard to the copper particle volume percentage. The thermal conductivity of the copper particles is much higher than that of the silicon base. The thermal conductivity of the silicon base without addition of any copper powder is 3 ± 0.3 W/m·k. It can be observed that the thermal conductivity of the thermal grease increases as the volume percentage of the copper particles increases. There is an overall rapid increase up to 20 vol. %, and the thermal conductivity is 7 ± 0.4 W/m·k. The currently commercialized thermal grease (RTC-6.5, made by 3rsys) is 6.5 W/m·k, and the maximum thermal conductivity of the manufactured thermal grease is higher. It then gradually increases after 20 vol. %. This can be attributed to the increase in the thermal conductivity as the amount of copper powder added increases and the thermal contact area inside the thermal grease increases. However, after reaching 20 vol. %, the thermal contact resistance increased as the silicon base layer was thinned between the copper particles resulting to a decrease in the thermal contact area.

The thermal conductivity when the copper particle volume percentage is 25 vol. % reaches 7 ± 0.3 W/m·k, but the viscosity of the thermal grease is not high, fluidity is not maintained, and stirring is not performed smoothly. It is considered that the copper powder is not uniformly dispersed, and thus the contact area between the particles is reduced and the thermal conductivity improvement is reduced.

The thermal conductivity of graphene mixed with copper powder increased up to about 0.6 W/m·K compared to graphene without copper powder, and thermal conductivity increased up to about 0.75 W/m·k for alumina.

In the study of Wei Yu et al. [29], the maximum thermal conductivity was about 3.45 W/m·K, while this paper confirmed the maximum thermal conductivity of 7 ± 0.3 W/m·K.

### 3.4. Thermal Conductivity of Thermal Grease

Graphene and alumina are materials with high thermal and electrical conductivity and are used as filler additives like copper. In particular, graphene is excellent as a filler for improving thermal conductivity. However, the proper area ratio of the additive is important. In order to prepare a thermal grease having excellent thermal conductivity, 5 vol. % of copper powder was mixed with 1 vol. % graphene and 1 vol. % alumina to improve thermal conductivity by increasing the contact volume ratio and surface area between particles.

Figure 8 shows the results of the thermal conductivity and thermal conductivity ratio measurement of the manufactured thermal grease. Samples of “A copper powder” stored for 6 months or longer and “B copper powder” stored within 6 days of the manufacture day show little difference in thermal conductivity ratio of 0.03 at 5 vol. % by volume. However, as the volume percentage increases, Rain increases. In the case of alumina, when the 5 vol. % copper is added to the pure alumina at 15 vol. %, the thermal conductivity is improved by approximately 20%.

In the case of graphene, the thermal conductivity was improved by about 24% when 5 vol. % copper was added at 15 vol. % compared to when pure graphene and silicone oil were mixed.

It is believed that the thermal conductivity of thermal grease of the composite material was increased by increasing the optimal packing volume ratio between particles.

## 4. Conclusions

In this study, commercially available thermal grease was prepared by mixing copper nano powders prepared by an electric line explosion method with graphene and alumina, respectively, on a silicon base. The thermal conductivity of each composite material thermal grease was improved, and the conclusions are as follows.

- As a result of observation by FE-SEM, it can be seen that the prepared nano-powder has a uniform spherical shape with an average size of 100 nm in radius. In addition, it was confirmed that the particle size volume ratio was 85:15.
- As a result of BEI observation after nano thermal grease production, it was confirmed that the nano powders were not agglomerated but evenly distributed.

As a result of the thermal conductivity measurement, it can be seen that the fresh copper powder has a maximum thermal conductivity ratio of 0.03 better than the old copper powder.

- Up to 15 vol. %, the upward trend of thermal conductivity increases steadily, while after 20 vol. %, the increase trend decreases. In addition, the increase rate of thermal conductivity from 0 to 5 vol. % and 10 to 15 vol. % is the largest.
- There is no significant difference in thermal conductivity between copper powder A and copper powder B, but there is a slight difference in thermal conductivity when other additives are added.

In conclusion, the thermal conductivity of the thermal grease mixed with graphene and copper powder was the best, and the value was about 7.5 W/m·k. In addition, when manufacturing the thermal grease, it is considered that the optimum volume ratio is up to 15 vol. **%** because the increasing tendency is significantly reduced at 20 vol. **%** or more. In the future, it is planned to be used as a heat sink for electronic devices.

## Figures and Tables

**Table 1:** Mixing conditions of copper nano powder.

Material	Voltage (V)	Number of Time	Wire Diameter (mm)	Wire Length (mm)
Copper	4500	2000	0.2	32

**Table 2:** Manufacturing conditions of thermal grease.

No.	Previous Thermal Grease (cc)	S/O (cc)	Volume Ratio (Vol. %)	Copper (g)
1	100	100	0	0
2	-	95	5	44.7
3	-	90	10	89.4
4	-	85	15	134.1
5	-	80	20	178.8
6	-	75	25	223.5
